# Routine 6-Week Outpatient Visit in Patients After Osteosynthesis of Lower Extremity Fractures: Is It Truly Necessary?

**DOI:** 10.3390/jcm15187124

**Published:** 2026-09-14

**Authors:** Stephanie Strauven-Heppner, Bryan J. M. van de Wall, Steffen Geuss, Lizzy Weigelt, Björn-Christian Link, Frank J. P. Beeres

**Affiliations:** 1 Faculty of Health Sciences and Medicine, University of Lucerne, 6002 Lucerne, Switzerland; bryan.vandewall@luks.ch (B.J.M.v.d.W.); steffen.geuss@luks.ch (S.G.); lizzy.weigelt@luks.ch (L.W.); bjoern-christian.link@luks.ch (B.-C.L.); frank.beeres@luks.ch (F.J.P.B.); 2 Department of Orthopedic and Trauma Surgery, Cantonal Hospital of Lucerne, 6004 Lucerne, Switzerland; 3 Department of General Surgery, GFO Clinics Troisdorf, 53840 Troisdorf, Germany

**Keywords:** fractures, lower extremity, osteosynthesis, follow-up, postoperative follow-up, postoperative X-ray

## Abstract

**Background/Objectives**: The rising fracture burden has strained healthcare systems, prompting a reassessment of standard practices. One of such practices—the six-week postoperative outpatient visit and X-ray for lower extremity fractures—remains standard, though its value in modern care is increasingly uncertain. **Methods**: This retrospective study examined patients with the most common lower extremity fractures (including proximal femur, femoral and tibia shaft and bi-/tri-/malleolar fractures) treated surgically at a Level 1 trauma center between January 2020 and March 2024. It focused on two outcomes on the six-week postoperative visit: Firstly, the incidence of radiographic abnormalities on X-rays taken—defined as discrepancies compared to intra- or immediate postoperative images—and their clinical relevance. Secondly, the incidence of deviations from local standard postoperative treatment and follow-up protocols. Abnormalities and deviations were classified as necessitating additional diagnostic tests, further therapeutic interventions, modifications to the standard postoperative regimen—including immobilization, weightbearing status, and permitted range of motion (ROM). **Results**: A total of 275 patients were included. Deviations from standard care, based on the outpatient visit as a whole, occurred in 9%, rarely requiring reinterventions. Six-week X-rays abnormalities were found in 6%. Of all X-rays, only 2% had a clinical consequence; of those, one patient was asymptomatic. **Conclusions**: These findings suggest that routine six-week follow-up visits and radiographs after surgical treatment of common lower limb fractures seldom result in changes in clinical management. While the 6-week visit may provide additional value through assessment of functional recovery, rehabilitation guidance, reassurance, and addressing practical concerns, these benefits need to be balanced against the limited clinical yield of routine hospital-based follow-up. These findings therefore raise the question of whether a routine 6-week hospital visit and radiograph are warranted for every patient, and whether alternative models of follow-up, including physiotherapist-led assessment with selective referral to the treating hospital, could provide a more targeted approach.

## 1. Introduction

The burden of fractures on healthcare systems has increased substantially in recent decades [1], prompting a reassessment of standard practices [2], driven largely by a growing and aging population with higher functional expectations [3]. This trend has contributed to rising healthcare costs, threatening the sustainability of modern healthcare systems. Efforts to reduce costs have primarily focused on optimizing diagnostics and treatments [4]. However, newer interventions often offer only marginal improvements over existing approaches, in contrast to decades past when medical advances represented substantial leaps forward. Consequently, this type of research has limited the potential to achieve major cost reductions. A more impactful approach may lie in critically evaluating routine practices. A perfect example is the standard scheduling of outpatient visits and radiographs at six weeks [5] (see Table 1) and three months for patients after lower extremity fractures. Although guidelines recommend this practice and surgeons routinely have been doing this in the past decades, we have stopped asking ourselves one (or two) simple question(s): why do we do it and is it still necessary in the current day?

Data on the utility of six-week outpatient visits and radiographs for patients surgically treated for lower extremity fractures are limited. A recent study on upper extremity fractures demonstrated minimal clinical value for these routine checkups [6]: radiographic abnormalities were rare, and when present, they were typically of little clinical significance. Complications were uncommon, and patients who did experience them were usually symptomatic. While similar findings might apply to lower extremity fractures, key differences—such as weightbearing function and a higher risk of infections—make direct extrapolation uncertain.

To address this gap, the present study aims to investigate the incidence of abnormal findings—both clinical and radiographic—during six-week postoperative outpatient visits and evaluate their clinical consequences in patients surgically treated for the most common lower extremity fractures. By assessing the medical necessity of these routine visits, this study aims to explore potential adaptations to postoperative care that could reduce the burden on healthcare systems while maintaining patient safety and quality of care.

## 2. Materials and Methods

### 2.1. Study Design and Population

This retrospective cohort study included patients that were treated between 1 January 2020 and 31 March 2024 in a level 1 trauma center in Switzerland. The hospital records were screened for all patients that underwent osteosynthesis for the most common lower extremity fractures including proximal femoral fractures, femoral shaft fractures, tibia shaft fractures and malleolar, bimalleolar and trimalleolar fractures. Patients with open fractures, periprosthetic fractures, arthroplasty/arthrodesis or who underwent revision surgery were excluded. The authors did not have access to information that could identify individual participants.

The study was carried out and reported according to the STROBE “Strengthening the Reporting of Observational Studies in Epidemiology” statement guidelines. Ethical approval has been granted (BASEC Nbr. 2025-01526).

### 2.2. Baseline Characteristics

Data on baseline characteristics were collected from the hospital records and included gender, age, ASA (American Society pf Anesthesiologists) grade, fracture type, interval between surgery and 6-week follow-up visit and type of osteosynthesis performed.

Fractures were classified according to the AO/OTA (ref) and subclassified for the ankle fractures. Types of osteosynthesis were categorized into nail or plate osteosynthesis.

### 2.3. Outcome

Two key outcomes were examined in this study. The first focused on the occurrence of abnormalities detected on the radiographs taken during the 6-week follow-up visit.

Abnormalities were identified as any discrepancies between the intraoperative (or immediate postoperative) radiographs and those obtained at the 6-week mark. Radiograph images were independently reviewed by two assessors to identify any abnormalities. If they could not reach a consensus, a third reviewer was consulted. When an abnormality was found, the hospital records were reviewed to assess its clinical significance. The clinical consequences were then classified based on whether they necessitated further diagnostics, additional interventions, changes to the standard postoperative immobilization, adjustments to weightbearing, or modifications to the permitted range of motion.

The second outcome of interest was the frequency of deviations from the standard local postoperative care and follow-up protocols as assessed during the 6-week outpatient visit (both clinical and radiological findings). If any deviations were identified, they were classified in the same manner as described for radiological abnormalities. The local standard postoperative care and follow-up protocols are outlined in Table 1.

### 2.4. Statistical Analysis

All statistical analyses were performed using the IBM SPSS statistics 30.0 Apple software version. For all demographic groups, descriptive statistics for clinical characteristics, treatment aspects and outcomes of interest per treated fracture were provided. Counts and percentages were calculated for categorical variables. For continuous variables, medians were used for the description (with interquartile range).

## 3. Results

### 3.1. Baseline Characteristics

**Figure 1:**
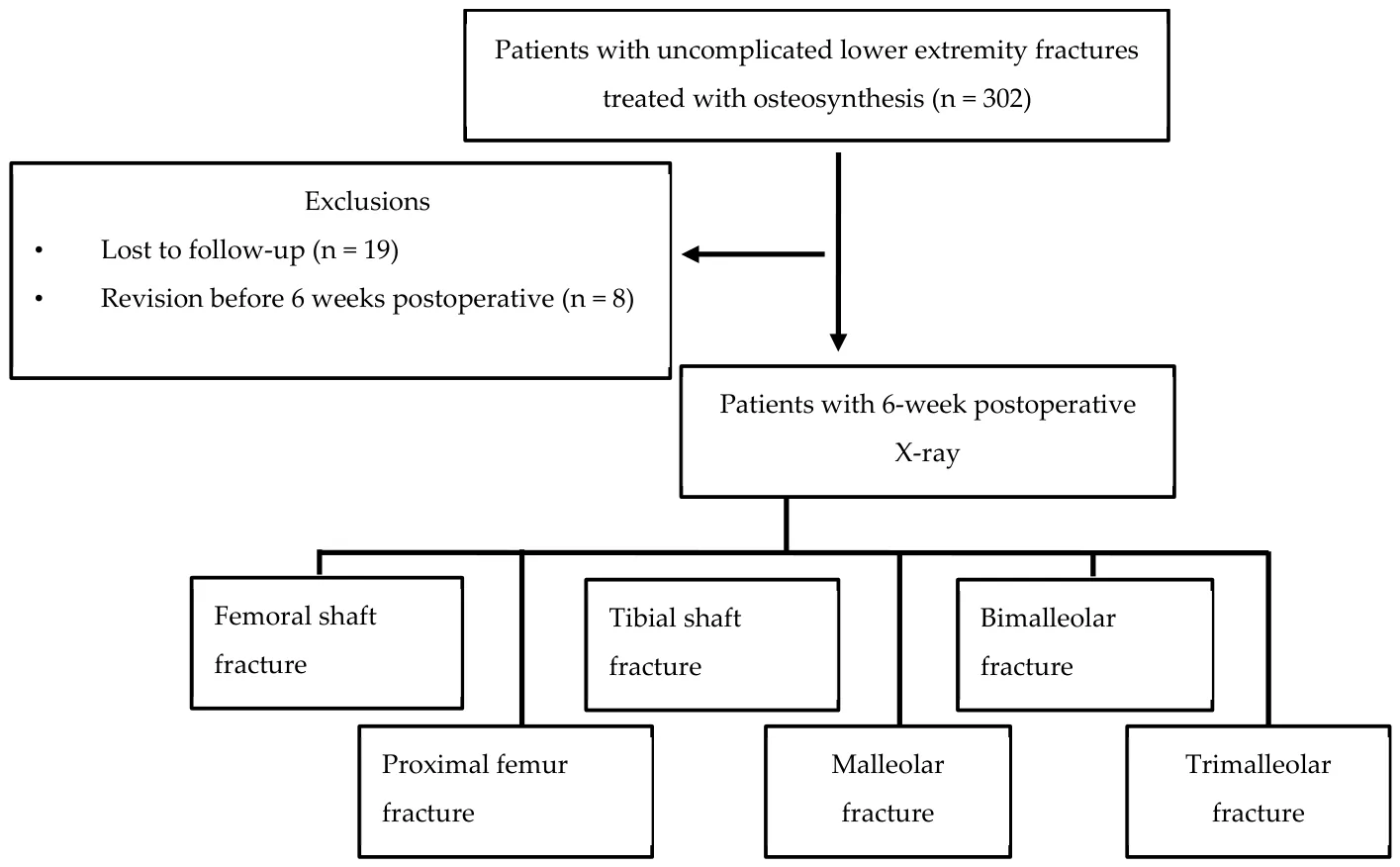
Patient sample.

A total of 302 patients with the lower extremity fractures of interest were screened according to the above-mentioned criteria. Nineteen of them were lost to follow-up and eight required surgical revision prior to the 6-week visit. A total of 275 patients were included for the final analysis (see Figure 1). All baseline and surgical characteristics are described in Table 2, Table 3 and Table 4. All X-ray abnormalities and deviations in standard aftercare are described in Table 5 and Table 6.

### 3.2. Outcomes According to Each Fracture

#### 3.2.1. Proximal Femoral Fracture

A total of eight (17%) abnormalities were found on the 6-week radiograph in patients with proximal femur fractures. In only one (2%) patient, this had a clinical consequence. The X-ray of this patient showed a cranial dislocation of the trochanter major fragment causing tractus iliotibialis syndrome. He received additional physiotherapy. The remaining X-ray abnormalities without any consequences included slight sintering of the fracture or femoral neck blade, dorsal tilt of the femoral head and progressive heterotopic ossification of the trochanter minor.

The outpatient visit led to a deviation from standard care in six (12%) patients, including the previously described case. Four patients all received additional physiotherapy due to inadequate progression in muscle strength of the quadriceps. One of them was also seen earlier in the outpatient clinic to check on progression. One patient was advised to prolong use of the crutches without any documented reason.

#### 3.2.2. Femoral Shaft Fracture

A total of two (5%) abnormalities were found on the 6-week radiograph in patients with femoral shaft fractures. One of the abnormalities led to operative reintervention; in this case, a loosened and migrated distal locking screw was removed. The other had no clinical consequences and included a heterotopic ossification cranially of the trochanter major without clinical symptoms.

In four (10%) patients, the outpatient visit led to a deviation from standard aftercare. This number included the previously described patient. One patient presented with pain in the trochanter major region and M. quadriceps shortening leading to intensification of physiotherapy. In the other two cases the partial weightbearing period was extended for reasons not described in the hospital records.

#### 3.2.3. Tibial Shaft Fracture

Only one (3%) abnormality was found on the 6-week X-ray in patients with tibial shaft fractures leading to a deviation from standard aftercare. The X-ray showed loosening of the distal locking screw. As the patient presented with correlating pain in this region, a reintervention with locking screw exchange was performed.

In five (15%) patients, the outpatient visit led to a deviation from standard care. Besides the previously described patient, four of these patients were advised to prolong partial weightbearing due to persisting pain in the fracture region or prolonged wound healing.

#### 3.2.4. Unimalleolar Fracture

Only one (2%) abnormality was found on the 6-week radiograph in patients with unimalleolar fractures. In this patient, a loosening of the syndesmotic screw was detected, leading to an additional visit after three weeks to check for further migration. Ultimately, the screw was left in place.

This was the only patient in whom the 6-week follow-up led to a deviation from standard aftercare.

#### 3.2.5. Bimalleolar Fracture

A total of two (4%) abnormalities were found on X-ray at the 6-week radiograph in patients with bimalleolar fractures, including a dislocated syndesmotic screw and incongruency of the joint lines. Clinically, the dislocated syndesmotic screw was palpable from the outside and caused pain upon external pressure; therefore, a screw removal was scheduled. For the patient with the incongruency of the ankle joint, an additional CT was planned, which showed a lateral talus shift, widening of the tibiofibular joint and loosening of the syndesmotic screw. Re-osteosynthesis and refixation of the syndesmosis was performed. Notably, the patient was asymptomatic at all times.

Including the previous patients, a total of five (10%) patients had deviations from standard aftercare. One patient had prolonged wound healing requiring extension of the partial weightbearing period. The second patient had a considerable limitation in the dorsal extension of the foot leading to intensification of physiotherapy. In the third patient, prolongation of immobilization was advised due to persistent swelling around the ankle.

#### 3.2.6. Trimalleolar Fracture

A total of two (4%) abnormalities were found on X-ray at the 6-week radiograph in patients with trimalleolar fractures. One of the abnormalities had a clinical consequence. A minor dislocation of the medial malleolus and the Volkmann fragment was observed after a plate osteosynthesis. Additionally, a necrotic wound area was seen at the lateral malleolar region. As a consequence, prolonged immobilization in a cast with daily wound care was initiated and another checkup appointment was scheduled two weeks later. In the second case, the syndesmotic screw migrated, leading to a change in the standard aftercare as an additional follow-up 6 weeks later.

In four (8%) patients, the outpatient visit led to a deviation from standard care, including the previously described patients. The other two patients presented with prolonged postoperative pain or swelling around the ankle. Each of them had difficulties with postoperative mobilization, leading to intensification of physiotherapy.

## 4. Discussion

### 4.1. Summary of Results

This retrospective study included 275 patients with the most common lower extremity fractures (proximal femur, femoral shaft, tibia shaft, uni-/bi-/trimalleolar) between January 2020 and March 2024 treated operatively in a level 1 trauma center. Abnormalities on X-ray at 6 weeks were found in 16 patients (6%). Out of all X-rays, it had clinical consequences only in six patients (2%), with five of those patients being symptomatic. Deviations from standard care based on the outpatient visit as a whole, occurred slightly more frequently in 25 patients (9%), and had a clinical consequence in 23 patients (7%) with reinterventions in four patients (1%), deviations from standard weightbearing in 10 patients (3%) and others, i.e., intensification of physiotherapy, in nine patients (3%).

### 4.2. Comparison with Previous Studies

To the best of our knowledge, only a few studies have been published to date, dealing with the topic of X-rays taken at the postoperative outpatient visit and their influence on deviations from standard care for lower extremity fractures.

Phelps’ study [7] reported that of 586 fractures, which only included fractures of the femur, tibia and ankle, 93 postoperative courses deviated from the norm due to radiographic findings alone. In 2% of the deviated cases, the responsible X-rays were taken in the direct postoperative period after surgery and during the partial/non-weightbearing period which generally corresponds with the first 6 weeks as investigated in the present study. It was pointed out that 69% of the total deviated 93 patients presented with symptoms, prompting the question of whether routine X-rays were necessary or should rather be limited to certain periods or to selective cases, for example, patients exhibiting symptoms.

Nair’s study [8] investigated over a 3-month postoperative period the contribution of X-rays after intramedullary nailing of tibia and femur fractures on postoperative management.

In 374 patients, 617 X-rays were taken, of which only nine (1.5%) contributed to a deviation from standard postoperative management, leading to the conclusion that X-rays in the first 3 months postoperatively do not result in changes to clinical management, a conclusion that our data also can support.

In summary, other studies support the findings of this study. However, those studies focused on specific fractures and only on the value of X-rays while this study also takes into account the clinical value of the 6-week control visit as a whole and a broader spectrum of fractures to the lower extremity.

### 4.3. Interpretation of Results and Clinical Implications

From a broader economic perspective, the findings suggest that routinely scheduling 6-week clinical and radiologic follow-up appointments may not be necessary. Most patients who required changes in their follow-up care showed symptoms indicating possible complications. While challenging to prove, it is reasonable to assume that, with proper postoperative instructions, these patients might have sought medical attention on their own even without a scheduled visit. However, completely eliminating routine follow-ups could result in missed complications, raising an important question: what level of risk is acceptable to lessen the strain on the healthcare system?

Importantly, risk tolerance varies between healthcare systems and cultural contexts. In the United States, orthopedic surgeons face a high risk of litigation, with nearly 99% of those aged 65 having experienced at least one malpractice lawsuit [9]. One of the leading causes of such claims is the failure to make timely diagnoses.

In contrast, malpractice claims are less frequent in many European countries. In cases of patient dissatisfaction, some countries even provide compensation for preventable harm without needing to prove negligence by the physician [10]. As a result, European healthcare systems tend to be less defensive and more lenient in their approaches compared to the U.S.

A thorough cost-effectiveness analysis could offer valuable insights into this issue. Still, the central dilemma remains: how much financial savings justify accepting the risk of overlooking complications? Regardless of the approach, cultural context plays a critical role in how such findings are interpreted.

Beyond the detection of radiographic or clinical abnormalities, the routine 6-week follow-up may have benefits that are less readily captured by clinical outcome measures. The consultation provides an opportunity to review functional recovery and range of motion, reinforce rehabilitation and home-exercise recommendations, and clarify the expected course of recovery. It may also facilitate communication between the patient, treating surgeon, and physiotherapist and allow practical issues, such as return-to-work planning, to be addressed. Furthermore, the reassurance provided by a scheduled clinical assessment may be valuable to both patients and clinicians, even when no change in treatment is required. These potential psychological and practical benefits should therefore be considered when evaluating the overall value of a routine 6-week visit. However, their impact on patient experience and satisfaction could not be assessed in the present study because of its retrospective design.

This study does not argue for the complete elimination of routine 6-week follow-up visits or X-rays, but rather aims to raise awareness about the potential to reduce these visits and X-rays to only what is truly necessary. A more selective approach could help optimize outpatient care without compromising patient safety. One strategy might involve more detailed postoperative instruction, empowering patients to monitor their own recovery and seek medical attention if they experience delayed progress or signs of complications. Alternatively, the 6-week follow-up could be performed by a physiotherapist. Physiotherapists may be well-positioned to identify signs of delayed or abnormal recovery and detect potential complications during routine rehabilitation. Patients with concerning findings could then be selectively referred to the treating hospital or orthopedic surgeon for further clinical and radiographic assessment. Furthermore, virtual/online follow-ups could be implemented to extend patient-adjusted scheduling. Walk-in clinics could offer advice in person, if symptoms arise. Different visit timings could be evaluated by the orthopedic doctor depending on the operation and patient compliance. Additionally, utilizing digital platforms to deliver follow-up information could be highly beneficial. Research shows that patients forget 40–80% of the information shared during medical consultations, and nearly half of what they do recall is often inaccurate [11]. Digital tools could reinforce key recovery guidelines, improve patient understanding, and encourage timely action when issues arise. All suggestions rely on good patient compliance.

### 4.4. Limitations

The research was conducted at a single medical center, which may limit the generalizability of the findings. Differences in surgical proficiency, patient populations, and healthcare practices across regions could influence the applicability of the results in other clinical settings. Additionally, while many European institutions follow the AO Foundation’s guidance for scheduling follow-up appointments and imaging—typically at intervals ranging from 3 to 8 weeks—the study site opted for a uniform 6-week interval for all fracture cases. Such variations in follow-up timing could affect the relevance of the findings for facilities with alternative scheduling protocols. The collected data did not include patient-reported outcome due to its retrospective design, limiting the study perspective to the doctor dimension. Lastly, the study was exploratory in nature, aimed at gaining initial insight into the frequency of deviations from standard aftercare procedures and radiographic anomalies. The relatively small sample size limits the statistical precision of the outcomes.

## 5. Conclusions

The findings of this explorative study suggest that routine 6-week follow-up visits and radiographs after surgical treatment of common lower limb fractures seldom result in changes in clinical management. While the 6-week visit may provide additional value through assessment of functional recovery, rehabilitation guidance, reassurance, and addressing practical concerns, these benefits need to be balanced against the limited clinical yield of routine hospital-based follow-up. These findings therefore raise the question of whether a routine 6-week hospital visit and radiograph are warranted for every patient, and whether alternative models of follow-up, including physiotherapist-led assessment with selective referral to the treating hospital, could provide a more targeted approach.

## Figures and Tables

**Table 1:** Local standard postoperative care and follow-up regimens.

Time Frame	Proximal Femur Fracture	Femoral Shaft Fracture	Tibial Shaft Fracture	(Mono) Malleolar Fracture	Bimalleolar Fracture	Trimalleolar Fracture
*First 6 weeks*						
Immobilization	None	None	None	Vacoped	Vacoped	Vacoped
ROM-Limitations	Free	Free	Free	Free	Free	Free
Weightbearing	Full	Full	15 kg	15 kg	15 kg	15 kg
X-ray	After 6 weeks	After 6 weeks	After 6 weeks	After 6 weeks	After 6 weeks	After 6 weeks
*After 6 weeks*						
Immobilization	None	None	None	None	None	None
ROM-Limitations	Free	Free	Free	Free	Free	Free
Planned reintervenions *	None	None	Dynamization **	Removal of syndesmotic screw	Removal of syndesmotic screw	Removal of syndesmotic screw
Weightbearing	Full	Full	Full (For plate osteosynthesis: 12 weeks 15 kg)	Full	Full	Full

* All patients are seen for a routine 6-week postoperative outpatient visit. ** Dynamization and/or removal of the syndesmotic screw was left to the discretion of the treating surgeon and considered part of standard operative care when specifically described as a planned secondary procedure in the report of the initial operation.

**Table 2:** Baseline characteristics per fracture.

Patient Characteristics	Total n = 275	Proximal Femur Fracture n = 48	Femoral Shaft Fracture n = 41	Tibial Shaft Fracture n = 33	Malleolar Fracture n = 52	Bimalleolar Fracture n = 52	Trimalleolar Fracture n = 49
Age, y, median	-	80 (51–97)	55 (18–92)	43 (16–70)	44 (17–82)	53 (21–92)	53 (22–92)
Sex, female, n (%)	159 (58%)	34 (71%)	23 (56%)	12 (36%)	26 (50%)	31 (60%)	33 (67%)
ASA							
classification,							
n (%)							
1–2	200 (75%)	18 (38%)	25 (61%)	31 (94%)	44 (85%)	42 (81%)	40 (82%)
3–4	68 (25%)	30 (62%)	14 (34%)	2 (6%)	6 (12%)	8 (15%)	8 (16%)
Type of							
fracture, n (%)							
A	105 (38%)	48 (100%)	29 (71%)	18 (55%)	5 (10%)	5 (10%)	0 (0%)
B	122 (45%)	0 (0%)	4 (10%)	3 (9%)	37 (71%)	37 (71%)	41 (84%)
C	48 (17%)	0 (0%)	8 (19%)	12 (36%)	10 (19%)	10 (19%)	8 (16%)
Polytrauma, n (%)		0 (0%)	0 (0%)	0 (0%)	0 (0%)	0 (0%)	0 (0%)

**Table 3:** Subclassification of malleolar fractures.

Subclassification	Total N = 153	Malleolar Fracture N = 52	Bimalleolar Fracture N = 52	Trimalleolar Fracture N = 49
Infrasyndesmotic Fibula fracture with medial malleolar fracture	5 (3%)	0 (0%)	5 (10%)	0 (0%)
Infrasyndesmotic Fibula fracture with medial malleolar fracture and Volkmann fragment	2 (1%)	0 (0%)	1 (2%)	1 (2%)
Transsyndesmotic Fibula Fracture without further damage	30 (20%)	30 (58%)	0 (0%)	0 (0%)
Transsyndesmotic Fibula fracture with medial malleolar fracture	26 (17%)	0 (0%)	26 (50%)	0 (0%)
Transsyndesmotic Fibula fracture with medial malleolar fracture and Volkmanns fragment	41 (27%)	0 (0%)	0 (0%)	41 (84%)
Transsyndesmotic Fibula fracture with Volkmanns fragment	11 (7%)	0 (0%)	11 (21%)	0 (0%)
Suprasyndesmotic Fibula fracture without further injuries	9 (6%)	9 (17%)	0 (0%)	0 (0%)
Suprasyndesmotic Fibula fracture with medial malleolar fracture	6 (4%)	0 (0%)	6 (12%)	0 (0%)
Suprasyndesmotic Fibula fracture with Volkmann fragment	3 (2%)	0 (0%)	2 (4%)	1 (2%)
Suprasyndesmotic Fibula fracture with medial malleolar fracture and Volkmann fragment	6 (4%)	0 (0%)	0 (0%)	6 (12%)
Medial malleolar fracture	13 (9%)	13 (25%)	0 (0%)	0 (0%)
Medial malleolar fracture with Volkmann fragment	1 (1%)	0 (0%)	1 (2%)	0 (0%)

**Table 4:** Treatment per fracture.

Treatment Aspects	Proximal Femur Fracture N = 48	Femoral Shaft Fracture N = 41	Tibial Shaft Fracture N = 33	Malleolar Fracture N = 52	Bimalleolar Fracture N = 52	Trimalleolar Fracture N = 49
Plate, n (%)	0 (0%)	3 (7%)	15 (45%)	8 (15%)	4 (8%)	3 (6%)
Nail, n (%)	48 (100%)	38 (93%)	18 (55%)	3 (6%)	0 (0%)	1 (2%)
Screws, n (%)	0 (0%)	0 (0%)	0 (0%)	10 (19%)	6 (12%)	0 (0%)
Plate and independent screws, n (%)	0 (0%)	0 (0%)	0 (0%)	31 (60%)	33 (63%)	40 (82%)
Plate and fibula nail, n (%)	0 (0%)	0 (0%)	0 (0%)	0 (0%)	0 (0%)	2 (4%)
Fibula nail and screws, n (%)	0 (0%)	0 (0%)	0 (0%)	0 (0%)	9 (17%)	3 (6%)
Interval between operation and 6-week X-ray visit, median in weeks	6.88	6.96	6.33	6.34	6.33	6.45

**Table 5:** Abnormalities on X-ray at 6-week checkup and their consequences.

	Total N = 275	Proximal Femur Fracture N = 48	Femoral Shaft Fracture N = 41	Tibial Shaft Fracture N = 33	Malleolar Fracture N = 52	Bimalleolar Fracture N = 52	Trimalleolar Fracture N = 49
Total X-ray abnormalities, n (%)	16 (6%)	8 (17%)	2 (5%)	1 (3%)	1 (2%)	2 (4%)	2 (4%)
Additional imaging	0 (0%)	0 (0%)	0 (0%)	0 (0%)	0 (0%)	0 (0%)	0 (0%)
Reintervention	4 (1%)	0 (0%)	1 (2%)	1 (3%)	0 (0%)	2 (4%)	0 (0%)
Deviation from standard weightbearing/ROM limitations	2 (1%)	1 (2%)	0 (0%)	0 (0%)	0 (0%)	0 (0%)	1 (2%)
Additional follow-up	2 (1%)	0 (0%)	0 (0%)	0 (0%)	1 (2%)	0 (0%)	1 (2%)

**Table 6:** Deviations from standard aftercare based on clinical/radiological findings in the outpatient visit at 6-week checkup.

	Total N = 275	Proximal Femur Fracture N = 48	Femoral Shaft Fracture N = 41	Tibial Shaft Fracture N = 33	Malleolar Fracture N = 52	Bimalleolar Fracture N = 52	Trimalleolar Fracture N = 49
Deviations from standard care, n (%)	25 (9%)	6 (12%)	4 (10%)	5 (15%)	1 (2%)	5 (10%)	4 (8%)
Additional imaging	0 (0%)	0 (0%)	0 (0%)	0 (0%)	0 (0%)	0 (0%)	0 (0%)
Reintervention	4 (1%)	0 (0%)	1 (2%)	1 (3%)	0 (0%)	2 (4%)	0 (0%)
Deviation from standard weightbearing/ROM limitations	10 (3%)	1 (2%)	2 (5%)	4 (12%)	0 (0%)	2 (4%)	1 (2%)
Additional follow-up	2 (1%)	0 (0%)	0 (0%)	0 (0%)	1 (2%)	0 (0%)	1 (2%)
Other (i.e., Physiotherapy)	9 (3%)	5 (10%)	1 (2%)	0 (0%)	0 (0%)	1 (2%)	2 (4%)

## Data Availability

The analyzed data was extracted from the hospital database of Luzern. All data supporting the findings of this study are available within the paper.

## Abbreviations

The following abbreviations are used in this manuscript:

AO	Arbeitsgemeinschaft für Osteosynthesefragen
ASA	American Society of Anesthesiologists
OTA	Orthopaedic Trauma Association
ROM	Range of Motion
